# Dissipation and Distribution of Prochloraz in Bananas and a Risk Assessment of Its Dietary Intake

**DOI:** 10.3390/toxics10080435

**Published:** 2022-07-29

**Authors:** Jiajian Huang, Sukun Lin, Jingtong Zhou, Huiya Chen, Shiqi Tang, Jian Wu, Suqing Huang, Dongmei Cheng, Zhixiang Zhang

**Affiliations:** 1 Key Laboratory of Natural Pesticide and Chemical Biology of the Ministry of Education, South China Agricultural University, Guangzhou 510642, China; 13923345997@163.com (J.H.); lsk951212@stu.scau.edu.cn (S.L.); chingtungchou@163.com (J.Z.); chya0929@163.com (H.C.); 13316149234@163.com (S.T.); 20212023016@stu.scau.edu.cn (J.W.); 2 College of Chemistry and Chemical Engineering, Zhongkai University of Agriculture and Engineering, Guangzhou 510225, China; hsuqing@zhku.edu.cn; 3 Department of Plant Protection, Zhongkai University of Agriculture and Engineering, Guangzhou 510225, China

**Keywords:** banana, prochloraz, high-performance liquid chromatography, dissipation dynamics, distribution, dietary risk assessment

## Abstract

Background: As a systematic fungicide, prochloraz is often used to control banana freckle disease, and it is significant to assess the safety and risk of prochloraz. Methods: The dissipation kinetics and distribution of prochloraz in bananas were measured by high-performance liquid chromatography (HPLC). Results: The results showed that the fortified recoveries in bananas were 83.01–99.12%, and the relative standard deviations (RSDs) were 2.45–7.84%. The half-life of prochloraz in banana peel (3.93–5.60 d) was significantly lower than it was in whole banana (8.25–10.80 d) and banana pulp (10.35–12.84 d). The terminal residue of prochloraz in banana fruits was below the maximum residue level (MRL, China) at pre-harvest intervals (PHI) of 21 d. Moreover, the residue of prochloraz in banana peel was always 1.06–7.71 times greater than it was in banana pulp. The dietary risk assessment results indicated that the prochloraz residue in bananas at PHI of 21 d was safe for representative populations. (4) Conclusions: We found that a 26.7% prochloraz emulsion oil in water (EW) diluted 1000-fold and sprayed three times under field conditions was safe and reliable, providing a reference for the safe application of prochloraz in bananas.

## 1. Introduction

Banana (*Musa acuminata*), the most important fleshy fruit around the world, is widely planted in China [1,2,3]. The banana contains a variety of water-soluble vitamins, including riboflavin, pyridoxine, folic acid, and vitamin B6 [4], making it an important food source for supplementing these vitamins needed by the human body [5,6,7]. Furthermore, banana by-products can be used in the production of food materials [8]. Their low price, high quantity, and ready availability can reduce the cost of feed if treated and utilized appropriately. However, many diseases may affect the quality of the banana before it is harvested [9,10,11]. Banana freckle is an economically significant disease on banana plantations; the disease causes pinhead-sized black spots on affected leaves and fruits [12]. For countries such as India which produce bananas for export, the infectious freckle disease makes the fruit less desirable [13]. In order to protect bananas from freckle disease, many fungicides are used, such as pyraclostrobin, tebuconazole, prochloraz, etc. [14].

**Figure 1:**
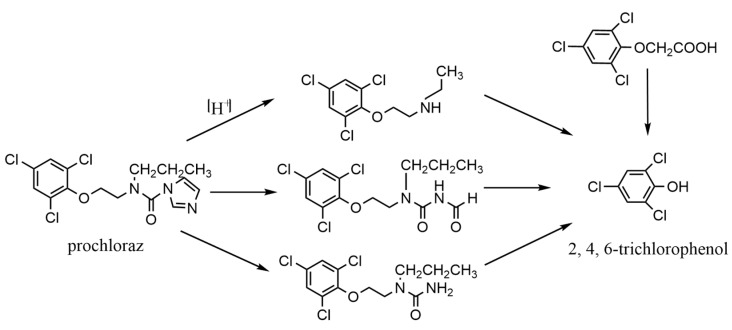
Degradation pathways of prochloraz.

Prochloraz (N-Propyl-N-(2, 4, 6-trichlorophenoxy) ethyl-imidazole-1-carboxamide) is a systemic imidazole fungicide and is widely used on banana for controlling freckle disease due to its high efficiency [15]. It can inhibit the synthesis of ergosterol and ultimately inhibit the growth of fungus [16]. It also has certain upward-conduction performance, which is widely used to control many plant diseases [17]. Hydrolysis is very common during breakdown of prochloraz, Figure 1 shows the degradation pathways of prochloraz. The product of the three degradation pathways of prochloraz is 2, 4, 6-trichlorophenol [18]. Therefore, it is necessary to detect 2, 4, 6-trichlorophenol when analyzing prochloraz residues. The sum of prochloraz and 2, 4, 6-trichlorophenol is considered as prochloraz [19]. In this study, all prochloraz was hydrolyzed to 2, 4, 6-trichlorophenol, and we then converted 2, 4, 6-trichlorophenol to the amount of prochloraz. According to the hydrolysis reaction of the chemometric method, 1 mol of prochloraz (molecular weight 376.7) hydrolyzes to yield 1 mol of 2, 4, 6-trichloroethane (molecular weight 197.5), and the conversion relationship between them is as follows:

1 (mg/kg) prochloraz = 0.525 (mg/kg) 2, 4, 6-trichlorophenol(1)

Prochloraz is a low-toxicity pesticide; however, its metabolite 2, 4, 6-trichlorophenol is characterized by stable structure and poor biodegradability, which can easily cause serious environmental pollution and is suspected to be a strong carcinogen [20]. Therefore, the toxicity of prochloraz to nontarget organisms at relevant concentrations has attracted people’s attention. The safety and risks of the indoor use of prochloraz on bananas were reported in a previous study [21]; however, the safety and risks of the field application of prochloraz on bananas are currently unclear. In a field application, it is necessary to understand the prochloraz residue’s dynamics on bananas to ensure the safety of the banana’s fruit and to protect the environment.

The objectives of this study were (a) to determine the dissipation dynamics of prochloraz residue on banana fruits under field conditions; (b) to analyze the prochloraz’s residual distribution and permeability of banana fruits; (c) to evaluate the exposure risk of prochloraz on banana fruits to representative populations in China. We aimed to provide a reference for the safe application of 26.7% prochloraz emulsion oil in water (EW) in bananas.

## 2. Materials and Methods

### 2.1. Chemicals and Reagents

Prochloraz standard (98%, CAS number 67747-09-5) and 2, 4, 6-trichlorophenol standard (98.0%, CAS number 88-06-2) were obtained from Aladdin (Shanghai, China). A 26.7% prochloraz emulsion oil in water (EW) was supplied by the Jinan Yinong Chemical Limited Company (Shandong, China). Ethyl acetate, acetonitrile (analytical grade), petroleum ether, formic acid, anhydrous sodium sulfate, pyridine hydrochloride, and anhydrous NaCl were obtained from the Yongda Chemical Reagent Factory (Tianjin, China). Acetonitrile and methanol (chromatographic-grade) were supplied by the Oceanpak Company (Gothenburg, Sweden). N-propyl ethylenediamine (PSA), ODS-C_18_, and graphitized carbon black (GCB) were obtained from the Agela Technologies Incorporated Company (Torrance, CA, USA). The stock standard solution of prochloraz and 2, 4, 6-trichlorophenol was prepared at 100 mg/kg by methanol (HPLC-grade) and stored in a refrigerator at −20 ± 1 °C.

### 2.2. Field Experimental Design

The field experiment was conducted in Guangzhou (113.56° E, 22.69° N, Guangdong province, China), Yuxi (101.58° E, 24.05° N, Yunnan province, China), and Lingao (109.65° E, 19.87° N, Hainan province, China) in 2021. The production soil in three test location was red loam, with 4.1% organic matter and a pH at 5.5 in Guangzhou, 3.4% organic matter and a pH at 5.7 in Yuxi, 3.5% organic matter and a pH at 4.5 in Lingao. The banana varieties planted in Guangzhou, Yuxi, and Lingao were the Guangdong banana no. 2, Zhongjiao no. 9, and Brazil banana, respectively. Each treatment (location) had 6 plots with a buffer zone of 1 m, and each plot had six trees. At each location, three plots were randomly selected to have prochloraz sprayed, and the remaining three plots served as the control area. We diluted 26.7% prochloraz (EW) 1000-fold (maximum recommended dosage) with water and sprayed the mixture on whole banana plants three times at 7 days after the previous spray. The water consumption was 900 L/ha. In the control areas, the bananas were not sprayed with prochloraz throughout the whole developmental stage. In order to research the dissipation dynamics of prochloraz and the terminal residues in different parts of the banana under field conditions, representative banana samples were acquired at random from every point at 0 (2 h), 1, 3, 5, 7, 14, 21, 28, and 35 d after the last spray. All banana samples were homogenized and stored in the refrigerator at −20 ± 1 °C.

### 2.3. Sample Preparation

The banana fruit samples were peeled with a knife to separate the peel and pulp, and then each was put in a centrifuge tube. We added 5.0 g samples of a whole banana, the peel, and the pulp to a disposable centrifuge tube. We then added 25.0 mL acetonitrile to the disposable centrifuge tube and extracted samples by ultrasonication for 1 h. Next, 5.0 g NaCl was added to the centrifuge tube and centrifuged at 4000 r/min for 5 min using the centrifugal machine. We placed 25 mL supernatant solution into a flask. The supernatant of the acetonitrile layer was aspirated and mixed with 2.0 g of anhydrous sodium sulfate before the pressure was reduced. We then concentrated the mixture to 3.0 mL and transferred it to a 20 mL stopper tube to be dried with nitrogen. Next, 1.0 g of pyridine hydrochloride was added into the stopper tube and hydrolyzed in an oil bath at 220–240 °C for 1.5 h; after allowing the mixture to cool naturally, we took the tube out and cleaned it twice with 10.0 mL of water, transferred the tube to the separation funnel, extracted the transferred reactant with 40 mL × 2 ethyl acetate, and discarded the inorganic phase. We combined the mixture with the organic phase, dehydrated it using anhydrous sodium sulfate, and then rotated it to evaporate the solution until it was dry. Next, the cleaning solution was extracted to a centrifuge tube, and 0.050 g C_18_, 0.050 g GCB, and 0.050 g PSA were added for purification. Next, 1500.0 μL of methanol (chromatographic grade) was added until complete dissolution. The final step was to filter the supernatant with a 0.22 μm filter membrane and analyze the samples using HPLC.

### 2.4. HPLC Analysis

A Shimadzu LC-20A HPLC (equipped with UV detector, Shimadzu Company of Japan, Kyoto, Japan) was used to analyze the 2, 4, 6-trichlorophenol. The chromatographic column was analyzed with an Agilent Eclipse XDB-C_18_ (4.6 mm × 250 mm, 5 μm) and with water: acetonitrile (35:65), as the mobile phase, consisted at a flow rate of 1 mL/min. The sample size, detection wavelength, and column temperature were 35 °C, 290 nm, and 10 μL, respectively.

### 2.5. Analytical Method Validation

Method validation studies were tested for the whole banana fruit, banana peel, and banana pulp, including the linearity, limit of detection (LOD), limit of quantitation (LOQ), and fortified recovery, to evaluate their accuracy and precision. The LOQ was defined as the minimum amount of the measured substance in the sample that could be quantitatively determined with certain accuracy, identified by multiple additive recovery tests [22]. The LOD was defined as the concentration with a S/N (the ratio of signal to noise) of three times [23].

### 2.6. Risk Assessment

For fruit consumption groups of different ages and genders, we calculated the dietary intake risk for prochloraz in bananas based on the groups’ specific body mass and fruit intake, combined with the terminal residue data in our study [24]. The dietary intake risk assessment included chronic and acute risks. The chronic dietary intake risk was assessed by calculating %ADI, and %ADI was calculated using Equations (2) and (3) [25]:

NEDI = P × STMR/BW(2)

%ADI = (NEDI/ADI) × 100%(3)

where NEDI (mg/kg/d) denotes the national estimated daily intake of prochloraz, P (kg/d) denotes the average daily fruit intake, STMR (mg/kg) denotes the median of terminal residual test under field condition, BW (kg) denotes body weight, and ADI (mg/kg/d) denotes the allowable daily intake of prochloraz [26]. The acute dietary intake risk was assessed by calculating %ARfD, and %ARfD was calculated using Equations (4) and (5) [27]:

ESTI = LP × HR/BW(4)

%ARfD = (ESTI/ARfD) × 100%(5)

where ESTI (mg/kg/d) denotes the acute dietary exposure, LP (kg/d) denotes the maximum daily fruit intake, HR (mg/kg) denotes the highest value of prochloraz in a terminal residual test under field conditions, BW denotes body weight (kg), and ARfD (mg/kg/d) denotes the acute reference dose [28].

If %ADI (%ARfD) > 100%, this indicates an unacceptable risk of chronic (acute) dietary intake of prochloraz from bananas; the greater that the %ADI (%ARfD) is, the greater the risk is. Conversely, the chronic (acute) dietary intake risk of prochloraz from bananas is acceptable if %ADI (%ARfD) ≤ 100% [29].

### 2.7. Data Analysis

The dissipation dynamics and half-life of prochloraz were calculated using Equations (6) and (7), respectively [30]:

C_t_ = C_0_e^−kt^ (6)

t_1/2_ = ln2/k (7)

where C_0_ is the initial residual concentration of prochloraz, C_t_ is the residual concentration of prochloraz at the time of t, t_1/2_ denotes the half-life of prochloraz, and k is the constant of the dissipation degradation rate [31].

The data were sorted using Excel, and a statistical analysis was performed using analysis of variance (ANOVA) with the SPSS 17.0 Statistical Package for Social Sciences (SPSS Inc., Chicago, IL, USA) [32]. The values of the half-life and terminal residue were expressed as mean values after statistical analysis, and data graphs were generated using GraphPad Prism 8 (GraphPad Software, San Diego, CA, USA).

## 3. Results

### 3.1. Method Validation

**Figure 2:**
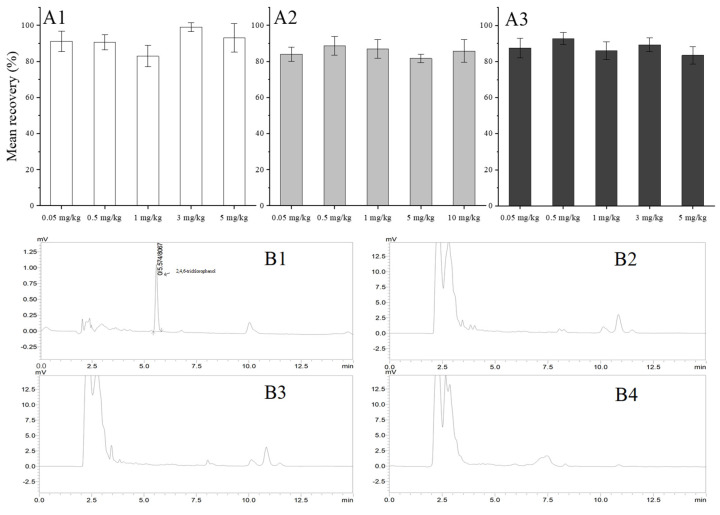
Mean recovery of 2, 4, 6-trichlorophenol in whole bananas (**A1**), banana pulp (**A2**) and banana peel (**A3**) and the chromatgram of of a standard of 2,4,6-trichorophenol at 0.5 mg/kg (**B1**), whole banana sample blank (**B2**), banana peel sample blank (**B3**), and banana pulp sample blank (**B4**). Mean recovery ± RSD (*n* = 5) are reported.

The content of prochloraz in each part of banana was determined by measuring the content of 2, 4, 6-trichlorophenol and converting it according to Equation (1). The standard linear regression formula and correlation coefficient for 2, 4, 6-trichlorophenol are y = 15499 x + 25.825 and 0.9999, respectively, indicating that 2, 4, 6-trichlorophenol standard kept a good linear relation in the concentration range of 0.05–10 mg/L. As shown in Figure 2(A1–A3), the results reflected that the mean recoveries of 2, 4, 6-trichlorophenol were 83.01–99.12% for the whole banana fruit, 84.00–88.70% for the banana peel, and 83.45–92.77% for the banana pulp. The RSDs ranged from 2.45–7.84% for the whole banana fruit, 3.93–6.32% for the banana peel, and 3.37–5.45% for the banana pulp, respectively. Some typical HPLC chromatograms of the 2, 4, 6-trichlorophenol standard solution at 0.5 mg/kg for the blank whole banana fruit, banana peel, and banana pulp are shown in Figure 2(B1–B4). It can be seen that there were no obvious endogenous interference peaks in the blank samples and no co-elution with other peaks. The experimental results indicated that the method has high precision and accuracy and is applicable for detecting 2, 4, 6-trichlorophenol residues in different varieties of banana fruits.

### 3.2. Dissipation of Prochloraz in Different Parts of Banana Fruits

**Figure 3:**
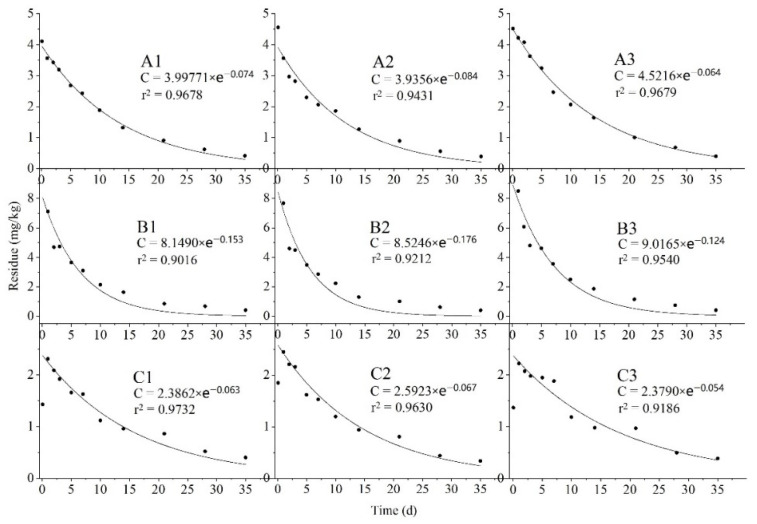
Dissipation equation of prochloraz residues in whole banana samples in Guangdong (**A1**), Hainan (**A2**), and Yunnan (**A3**); dissipation equation of prochloraz residues in banana peel samples in Guangdong (**B1**), Hainan (**B2**), and Yunnan (**B3**); dissipation equation of prochloraz residues in banana pulp samples in Guangdong (**C1**), Hainan (**C2**), and Yunnan (**C3**).

Figure 3 shows the dissipation equations and correlation coefficients of prochloraz in different parts of banana fruits collected in Guangzhou (Guangdong province), Lingao (Hainan province), and Yuxi (Yunnan province) under field conditions. It can be seen that the prochloraz residue in different parts of banana continued to decrease. The prochloraz residual amounts of the banana pulp samples were much lower than those of the whole banana fruits and banana peel. In addition, the residue of prochloraz in banana pulp never exceeded more than the MRL (5 mg/kg) stipulated by China.

**Figure 4:**
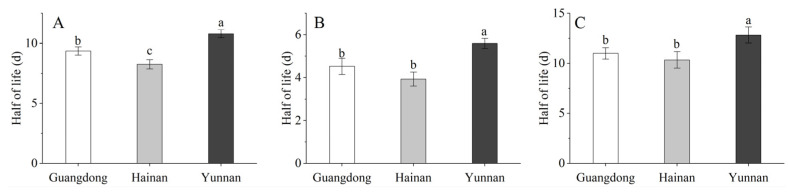
Half-life of prochloraz in a whole banana (**A**), banana peel (**B**), and banana pulp (**C**). Note: data are presented as mean ± SE (*n* = 3), with different lowercase letters in the same chart indicating significant differences at *p* < 0.05 using Duncan’s range test.

As shown in Figure 4, the prochloraz half-lives in different parts of the banana fruits from three different regions were 8.25–10.80 d for the whole banana, 3.93–5.60 d for the banana peel, and 10.35–12.84 d for the banana pulp. The prochloraz half-lives in different parts of banana fruits were as follows: Hainan > Guangdong > Yunnan. The prochloraz half-lives in the different pulps were significantly longer than those in the peels under the same conditions, which indicated that prochloraz was more easily degraded in peels than in the pulp. We confirmed that the half-lives of prochloraz in the collected whole bananas from the three southern provinces of China were significantly different from each other (*p* < 0.05). Furthermore, the prochloraz half-life in the banana peel from Yunnan was significantly different from that of the prochloraz half-life in the banana peel and pulp collected in Guangdong and Hainan (*p* < 0.05).

### 3.3. Prochloraz Terminal Residues in Different Parts of Banana Fruits

**Figure 5:**
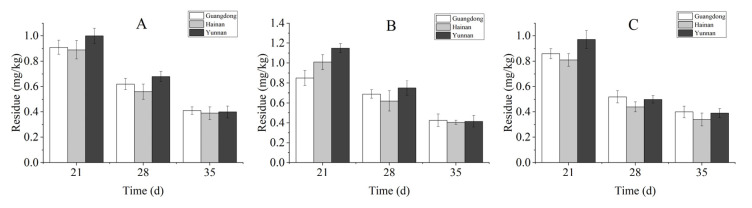
The terminal residues of prochloraz in a whole banana (**A**), banana peel (**B**), and pulp (**C**) from three different regions; data are presented as mean ± SE (*n* = 3).

The prochloraz terminal residues in different parts of the banana fruit from the three southern provinces of China were determined at 21, 28, and 35 d. As shown in Figure 5, there was no significant difference in the prochloraz residues in the three southern provinces of China. For the banana peel, the residual amounts of prochloraz were higher than those in the whole banana, and the residual amounts of prochloraz in the banana pulp were always less than the MRL (China) during the entire growth phase of the banana. In the three southern provinces of China, the prochloraz residual levels at 21 d were 0.89–1.00 mg/kg for the whole banana, 0.98–1.45 mg/kg for the banana peel, and 0.70–0.86 mg/kg for the banana pulp.

### 3.4. Prochloraz Distribution and Permeability in Different Parts of Banana Fruits

**Figure 6:**
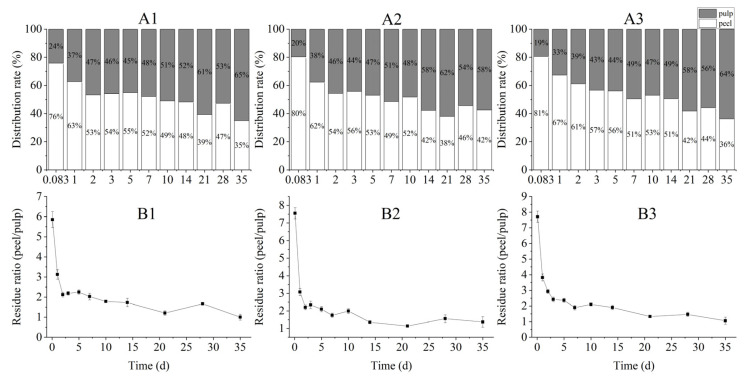
Distribution rate of prochloraz on the banana peel and pulp from the bananas from Guangdong (**A1**), Hainan (**A2**), and Yunnan (**A3**); residual prochloraz ratio of the banana peel to pulp from Guangdong (**B1**), Hainan (**B2**), and Yunnan (**B3**). Data are presented as mean ± SE (*n* = 3).

The prochloraz residues in the banana peel and pulp from the three southern provinces of China were measured, and their ratios were computed to measure the prochloraz permeability and distribution in the banana peel and pulp. The results indicated that prochloraz has great diffusivity in banana fruits (Figure 6A). The prochloraz content in the banana peel compared with that of the whole banana was 24–65% for the Guangdong samples, 20–62% for the Hainan samples, and 19–64% for the Yunnan samples. The prochloraz was mainly absorbed by the banana peel after the initial. As time went on, the proportion of prochloraz gradually decreased in the banana peels and increased in the banana pulps. At 7 d, the content of prochloraz in the banana peel was approximately equal to the prochloraz content in the banana pulp. After 10 d, the content of prochloraz in the banana peel was less than the prochloraz content in the banana pulp. As shown in Figure 6B, under field conditions, the residual amount of prochloraz in the banana peel was 5.85–7.71 times as much as the residual amount of prochloraz in the pulp at 2 h. After the last spray, the residue ratio for the peel to pulp began to degrade. Since prochloraz takes longer to dissipate in the pulp than it does in the peel, the proportion of prochloraz gradually decreased in the banana peels and increased in the banana pulp as time went on.

### 3.5. Exposure Risk Assessment

Dietary intake risk is related to food intake and body weight, which varies in different populations. As shown in Table 1, in this study, the six typical groups were divided to assess the chronic and acute dietary intake risk of prochloraz in banana pulp. The ADI and ARfD of prochloraz was 0.01 and 0.1 mg/kg/d, respectively. The terminal residue data at the pre-harvest interval (PHI) of 21d was determined, and the results showed that the STMR and HR in three regions were 0.93 and 1.00 mg/kg, respectively. The %ARfD was much less than 100%, and the six groups of population had risks of 24.07–25.33% for 2–4-year-olds, 8.43–9.70% for 18–30-year-olds, and 8.32–9.40% for 60–70-year-olds. The %ADI was also less than 100%, and the six groups of population had risks of 28.82–30.81% for 2–4-year-olds, 6.43–9.35% for 18–30-year-olds, and 5.13–5.94% for 60–70-year-olds. Children in the 2–4-year-old age group were most at risk. Furthermore, women in all groups had a greater risk of exposure than men. In general, the risk of the population was as follows: 2–4 years old > 18–30 years old > 60–70 years old. For prochloraz, the average %ARfD was <25.33%, and the average %ADI was < 30.81%, which were both significantly less than 100%. The results indicated that the chronic and acute dietary intake risk of prochloraz was within the acceptable level for China.

## 4. Discussion

Contamination of crops by pesticide residues is an ongoing challenge, and the rate of dissipation after the application of pesticides is an important factor and useful tool for evaluating residues’ behavior [33]. To evaluate the safety of prochloraz used in controlling banana freckle disease in the field, the dissipation and distribution of prochloraz in different parts of banana fruits were detected by HPLC. The results showed that the dissipation rates of prochloraz in different parts of banana fruits were significantly different and that prochloraz degraded more easily in the peel than in the pulp; moreover, the dissipation rates of the same part of the banana from different locations were also significantly different (Figure 4). There are many factors that affect the degradation of pesticides in crops, such as light, temperature, humidity, pH, etc. [34]. Therefore, environmental factors such as light and temperature are likely to be the reasons for the significant differences in the degradation rate of prochloraz. The terminal residue results showed that 21 days after the last application, the residues of prochloraz in banana fruits were below the MRL in China (Figure 5), which indicated that the terminal residual values of 26.7% prochloraz emulsion oil in water (EW) diluted 1000-fold and sprayed three times under field conditions were in line with the standard. The distribution results show that prochloraz has a certain permeability between the peel and pulp of bananas (Figure 6), which should be kept in mind when applying prochloraz in the field. Based on the terminal residues and related dietary data, the dietary intake risks of prochloraz in bananas were further assessed (Table 1). The dietary intake risk results indicated that the dietary intake risk of prochloraz in bananas was at an acceptable intake level. Overall, the results of this work indicate that 26.7% prochloraz emulsion oil in water (EW) diluted 1000-fold and sprayed three times under field conditions is safe and reliable and provides a scientific basis for the safe application of prochloraz on bananas under field conditions.

## Figures and Tables

**Table 1:** Dietary intake assessment of prochloraz in bananas under field conditions.

Age	Sex	BW (kg)	P (g/Day)	LP (g/Day)	ESTI [mg/(kg·d)]	%ARfD (%)	NEDI [mg/(kg·d)]	%ADI (%)
2–4	M	14.1	43.7	339.4	0.0241	24.07	2.88 × 10^−3^	28.82
	F	13.4	44.4	339.4	0.0253	25.33	3.08 × 10^−3^	30.81
18–30	M	60.5	41.8	510.2	0.0084	8.43	6.43 × 10^−4^	6.43
	F	52.6	52.9	510.2	0.0097	9.70	9.34 × 10^−4^	9.34
60–70	M	61.3	33.8	510.2	0.0083	8.32	5.13 × 10^−4^	5.13
	F	54.3	34.8	510.2	0.0094	9.40	5.96 × 10^−4^	5.96

Note: M, male; F, female; BW, body weight; P, average daily fruit intake; LP, maximum daily fruit intake.

## Data Availability

Not applicable.
